# Profiling of Metabolic Differences between Hematopoietic Stem Cells and Acute/Chronic Myeloid Leukemia

**DOI:** 10.3390/metabo10110427

**Published:** 2020-10-26

**Authors:** Byung Hoo Song, Su Young Son, Hyun Kyu Kim, Tae Won Ha, Jeong Suk Im, Aeli Ryu, Hyeji Jeon, Hee Yong Chung, Jae Sang Oh, Choong Hwan Lee, Man Ryul Lee

**Affiliations:** 1 Soonchunhyang Institute of Medi-Bio Science (SIMS), Soon Chun Hyang University, Cheonan 31151, Korea; thdqudgn@naver.com (B.H.S.); hyunkyu8505@naver.com (H.K.K.); htw5200@gmail.com (T.W.H.); vjvmf625@naver.com (J.S.I.); 2 Department of Bioscience and Biotechnology, Konkuk University, Seoul 05029, Korea; syson119@naver.com; 3 Department of Obstetrics and Gynecology, Soonchunhyang University Cheonan Hospital, Chungcheongnam-do 31151, Korea; bestal@naver.com (A.R.); hjjeon@schmc.ac.kr (H.J.); 4 Department of Microbiology, College of Medicine and Department of Biomedical Science, Graduate School of Biomedical Science and Engineering, Hanyang University, Seoul 04763, Korea; hychung@hanyang.ac.kr; 5 Department of Neurosurgery, College of Medicine, Soonchunhyang University, Cheonan Hospital, Cheonan 31151, Korea; 6 Research Institute for Bioactive-Metabolome Network, Konkuk University, Seoul 05029, Korea

**Keywords:** acute myelogenous leukemia, chronic myelogenous leukemia, hematopoietic stem cells, metabolome, THP-1, U937, KG-1, K562

## Abstract

Although many studies have been conducted on leukemia, only a few have analyzed the metabolomic profiles of various leukemic cells. In this study, the metabolomes of THP-1, U937, KG-1 (acute myelogenous leukemia, AML), K562 (chronic myelogenous leukemia, CML), and cord blood-derived CD34-positive hematopoietic stem cells (HSC) were analyzed using gas chromatography-mass spectrometry, and specific metabolic alterations were found using multivariate statistical analysis. Compared to HSCs, leukemia cell metabolomes were found to have significant alterations, among which three were related to amino acids, three to sugars, and five to fatty acids. Compared to CML, four metabolomes were observed specifically in AML. Given that overall more metabolites are present in leukemia cells than in HSCs, we observed that the activation of glycolysis and oxidative phosphorylation (OXPHOS) metabolism facilitated the incidence of leukemia and the proliferation of leukemic cells. Analysis of metabolome profiles specifically present in HSCs and leukemia cells greatly increases our basic understanding of cellular metabolic characteristics, which is valuable fundamental knowledge for developing novel anticancer drugs targeting leukemia metabolism.

## 1. Introduction

Leukemia, a type of hematologic malignancy, occurs when immature white blood cells grow abnormally. Leukemia is known to be related to carcinogenic genes, chromosomal abnormalities, or bone marrow damage caused by viruses, radiation, or chemicals. It can be acute or chronic, depending on its progress pattern. Additionally, it can be myeloid or lymphocytic in nature, depending on the type of white blood cells affected. Generally, acute leukemia occurs when immature hematopoietic stem cells (HSC) develop into malignant tumor cells, whereas chronic leukemia occurs when partially matured hematopoietic cells are transformed [1,2,3]. Similar to other cancers, leukemia cells show obvious genetic variation and reprogram nutrition acquisition and metabolic pathways to meet the demands for bioenergy, biosynthesis, and oxidation-reduction [4]. Generally, to meet the increased nutritional demand, cancer cells undergo metabolic reprogramming of ATP via conversion of pyruvate to lactate rather than channeling it to the tricarboxylic acid (TCA) cycle and oxidative phosphorylation (OXPHOS) in mitochondria [5,6]. However, recently, aerobic glycolysis and OXPHOS metabolism have been found to be actively involved in cancer cell growth and division [7]. Hence, each cell has a unique metabolic mechanism depending on cell fate, and thus, has a different type of metabolome. Therefore, metabolic profiling research has drawn much attention as an approach for immediate detection of dynamic cellular alteration and status, such as oncogenesis.

The metabolome is a set of metabolites that can be used to quantify phenotypes in cells, tissues, and diseases [8]. Through metabolome profiling, it is possible to provide basic physiological information on phenotype expression independently or in combination with gene expression data. It is also possible to identify, quantify, and systematically determine the shift of metabolomes in cells or tissues, which helps in providing a better understanding and re-analysis of metabolome networks in association with the physiologic and pathologic states of the metabolome group. It further allows for the elucidatation of disease-specific metabolome alterations in the network model. By detecting and defining the main metabolic changes in a disease state and comparing them with the metabolomic profile in the normal state, it is possible to identify the cause of disease [9,10]. In particular, the detection of alterations in a few metabolomes allows early diagnosis of disease. Further, metabolome profiling-based classification makes it easier to identify diagnostic and other biomarkers through a clustering pattern analysis. With metabolome-directed disease detection, especially the detection of a new metabolic mechanism, which may be the direct cause of a disease, the challenges related to the medicinal efficacy and adverse effects of existing drugs can be overcome, and new drugs for intractable diseases can be developed.

Unlike normal cells, cancer cells metabolize glucose by glycolysis rather than by producing ATP via further oxidative phosphorylation. Even in the presence of oxygen, many cancer cells produce ATP by abnormally depending on glycolysis. This is known as the Warburg effect (a process called aerobic glycolysis), the main characteristic of cancer cell metabolism [11]. This metabolic alteration resulting from DNA mutation does not mean that the cancer cell lacks respiratory capacity. Via aerobic glycolysis, intermediates in the process are converted to biosynthetic pathways, thereby generating nucleotides, lipids, and amino acids required by the fast proliferating cells. Therefore, metabolic alteration is considered to be necessary for cell growth and division. It is known that metabolic alterations in cancer cells inhibit immune responses against them and help activate oncogenes [12]. Since the Warburg effect is a critical change during tumorigenesis, it is important to accurately determine the metabolic mechanism of cancer cells to develop anticancer drugs. In addtion the oxidative phosphorylation in some cancer cells, including leukemias, lymphomas, pancreatic ductal adenocarcinomas, high OXPHOS subtype melanomas, and endometrial carcinomas, fails to be suppressed and cancer growth continues unabated [7].

Metabolomic profiling of tumor cells helps predict a patient’s present condition and changes that may occur in the future. Therefore, metabolomic reprogramming can be applied for oncotherapy [13]. In this study, gas chromatography was used to determine the metabolome expression in cell lines derived from acute myelogenous leukemia (AML), chronic myelogenous leukemia (CML), and cord blood (V-derived CD34 positive hematopoietic stem cells (HSCs) and to select a common metabolome between AML and CML for the identification of novel putative diagnostic biomarkers for leukemia.

## 2. Results

### 2.1. Metabolic Differences between HSCs and Leukemia Cell Lines

**Figure 1:**
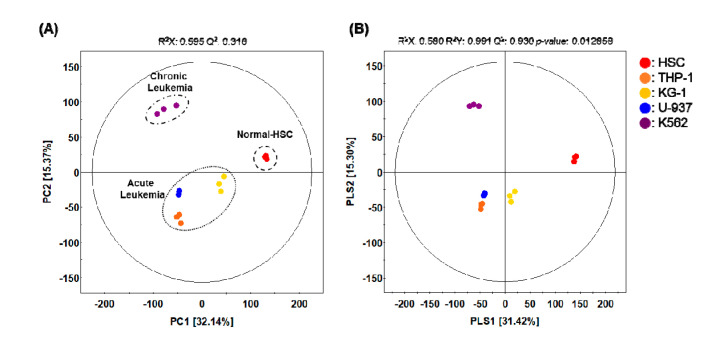
(**A**) Principal component analysis (PCA) and (**B**) partial least squares-discriminant analysis (PLS-DA) score plot derived from the GC-TOF-MS datasets of four different leukemia cell lines and one hematopoietic stem cells (HSC) line. (●, HSC; ●, THP-1; ●, KG-1; ●, U-937; ●, K562).

To analyze the specific metabolites of leukemia cells, three AML cell lines and one CML cell line were purchased from the American Type Culture Collection (ATCC). These cells, alongside normal blood cells and cord blood-derived HSCs, were cultured individually. Samples analyzed using GC-TOF-MS were used for further multivariate analysis of each feature. After that, by comparing the integrated metabolites, the relative abundance of metabolites for each cell population was quantified. We performed principal component analysis (PCA) and partial least squares-discriminant analysis (PLS-DA) to develop a visual plot for evaluating differences and consistencies in the metabolite profiles of four leukemia cell lines and HSC. The principal component (PC) scores used for PCA plotting increased for different cell types. Our PCA plot indicates that the normal control HSCs were clearly clustered from the leukemia cell line groups (THP-1, U-937, KG-1, and K562) along with PC1 (32.14%). Along with PC2 (15.37%), the normal control HSC and acute leukemia cell lines were clearly clustered from the chronic leukemia cell line groups (K-562) (Figure 1A). PLS-DA with model values of R^2^X_(cum)_ = 0.580, R^2^Y_(cum)_ = 0.930, and Q^2^_(cum)_ = 0.930 indicated that the fitness and prediction accuracy of the model were similar to the PCA results (Figure 1B). The quality of the model was evaluated by cross-validation analysis (*p* = 0.012858). PCA and PLS-DA showed obvious differences in the metabolite profiles of these cell types. Similar metabolite profiles between the cell types indicate that they are closely related in their metabolic properties, and hence, cell fate.

### 2.2. Hierarchical Clustering between HSCs and Leukemia Cell Lines

To select the metabolites responsible for the differences observed in Section 2.1, variable importance to projection (VIP) values > 0.7 of PLS–DAs were used. The VIP value is an important parameter for detecting potential biomarker candidates and possible pathways, including those involved in diseases that reflect the correlation of the metabolites with different biological states. For evaluating statistical significance, *p* < 0.05 derived from the one-way ANOVA was applied [14,15]. Selected metabolites were identified by comparing MS fragment patterns with commercial standard compounds and various databases, including the National Institutes of Standards and Technology (NIST) library, the Human Metabolome Database (HMDB, http://www.hmdb.ca/), and Wiley 9 [14,15]. Detailed information regarding these metabolites is presented in Table 1. A total of 43 metabolites, including five organic acids, eight amino acids, five sugars/sugar alcohols, eight fatty acids/lipid, three electron transport chains, and 14 unknown metabolites, were identified that differed significantly among the experimental groups.

**Figure 2:**
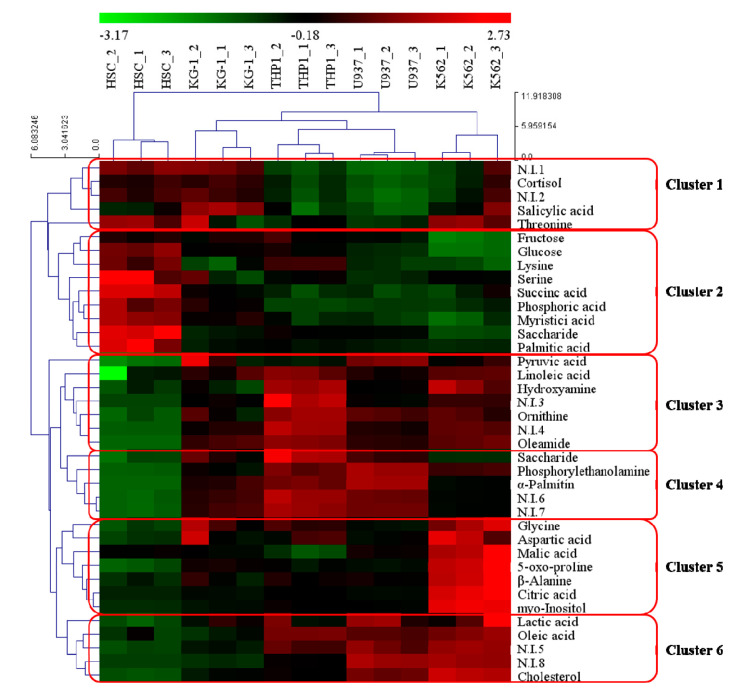
Heatmap showing hierarchical clustering analysis of metabolomic changes among four different leukemia cell lines and a normal hematopoietic stem cell (HSC) line. Metabolites were selected by VIP value > 0.7 and *p* value < 0.05. The rows display the metabolites, and the columns represent the cell lines. The colored squares (blue-to-red) represent fold changes normalized by averaging each metabolite of four different leukemia cells and a normal HSC line. The color scheme is as follows: lower limit value, −3.17 (green); middle limit value, −0.18 (black); upper limit value, 2.73 (red).

Our findings demonstrate the major differences in the metabolomic profiles of leukemia cells and normal HSC. As metabolite differences between these cells may have significant phenotypic consequences and include biomarkers for leukemia, we identified metabolites that differed between these different cell types. To obtain comprehensive metabolite accumulation patterns in our experimental cell groups, metabolites were organized by hierarchical clustering analysis (HCA; Figure 2), which revealed six clusters. The six clusters were based on the metabolites that were distinctively expressed in each cell type. Cluster 1 contained metabolites, such as threonine, cortisol, and salicylic acid, with relatively higher accumulation in HSCs, KG-1, and K562 cells. Cluster 2 was specifically expressed in HSCs and included fructose, lysine, phosphoric acid, succinic acid, myristic acid, glucose, saccharide, serine, and palmitic acid. Cluster 3 included metabolites such as pyruvic acid, linoleic acid, hydroxylamine, ornithine, and oleamide, which were specifically expressed in leukemia cell lines. Metabolites such as saccharide, phosphorylethanolamine, and alpha-palmitin from Cluster 4 were expressed in THP-1 and U937 cells. The metabolites belonging to Clusters 3 and 4 were relatively highly expressed compared to HSC, and Cluster 4 is a metabolite that was relatively highly expressed in THP-1 and U937 cells. Cluster 5 was specifically expressed in K562 cells as an AML and included glycine, aspartic acid, malic acid, 5-oxo-proline, beta-alanine, citric acid, and myo-inositol. Metabolites, such as lactic acid, oleic acid, and cholesterol from Cluster 6 were expressed in U937 and K562 cells. The HCA also grouped these samples separately according to the metabolic status, which suggests that differences in cell metabolite characteristics between leukemias and normal HSCs may reflect the reprogramming of metabolic systems during disease development.

### 2.3. Metabolic Differences Observed between HSCs and Leukemia Cell Lines Suggest Novel Putative Metabolic Biomarkers

**Figure 3:**
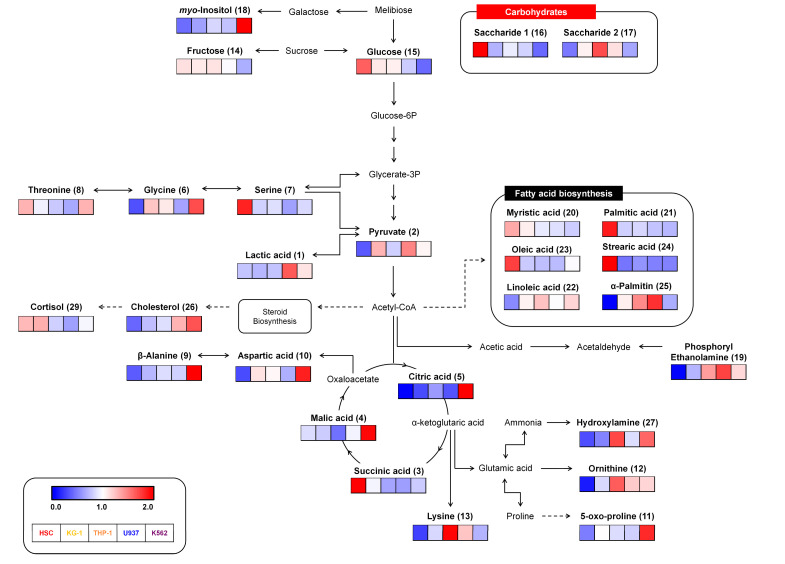
Constructed metabolic pathway showing relative metabolite contents for four different leukemia cell lines and a normal hematopoietic stem cell (HSC) line. The pathway was modified from the Kyoto Encyclopedia of Genes and Genomes (KEGG) database (http://www.genome.jp/kegg/). The colored squares (blue-to-red) represent relative metabolite abundance in the five cell lines.

Following the metabolomic analysis of HSC control and leukemia cell lines, various metabolites were selected as candidate biomarkers by multivariate analysis, and we generated a metabolic pathway to show the distribution and the relationships among these metabolites (Figure 3). These metabolites belong to pathways relating to amino acids, carbohydrates, and fatty acid biosynthesis. The relative levels of the metabolites were dramatically different between normal HSC control and leukemia cell lines. Carbohydrate metabolism linked to glucose and saccharide appeared to be upregulated in HSCs but downregulated in leukemia cell lines. In contrast, the metabolic pathways related to amino acid biosynthesis (glycine, aspartic acid, ornithine, lysine, 5-oxo-proline, and beta-alanine) were upregulated in leukemia compared to HSC. However, unlike other amino acid metabolites, serine was relatively downregulated in leukemia cell lines. Significant induction of these compounds (pyruvate, lactic acid, citric acid, and malic acid) coupled with intermediate glucose metabolism contents in leukemia cell lines suggest their collective contribution to leukemia development. However, normal HSCs showed higher levels of glucose in cellular metabolites than leukemia cell lines. Therefore, from these data, we suspected that glucose consumption might enhance leukemia cell metabolism to maintain the cancerous phenotype by increasing the levels of glycolysis and TCA intermediate metabolites to meet the heightened energy demands.

## 3. Discussions

The metabolome refers to the collection of all low molecular weight (10–1000 Da) metabolites in a biological cell, tissue, organ, or biological fluid. Metabolomes are produced as the final stage of biological processes and help to maintain cellular homeostasis. They are also useful for monitoring systemic changes in a living organism that cannot be understood via gene expression and proteome alteration studies alone. Metabolomic profiles provide actual snapshots of physiological conditions within biological systems by establishing a network of low molecular metabolites influenced by various genetic, physiological, pathological, or environmental factors. Thus, a comprehensive analysis of metabolomes can highlight the variations observed with physiological and disease states and may help to elucidate the basis for the observed differences. This offers valuable information for investigating the biological mechanisms influencing disease phenotypes. Accordingly, metabolome-based biomarkers can help identify specific phenotypes and serve as primary markers for determining the mechanism and basis for vital phenomena [16,17]. Here, we profiled the metabolomes of three types of AML-derived cell lines (TH-1, KG-1, and U937 cells) and one CML-derived cell line (K562), and compared our results with those obtained from normal HSCs. We defined the specifically expressed metabolomes in each cell line, verified the characteristics of each leukemia cell line, and proposed potential biomarkers. Following the metabolomic analysis of leukemia cell lines and normal HSCs, various metabolites and metabolic pathways were evaluated via multivariate analysis to identify candidate biomarkers (Figure 1 and Figure 2). Further, we linked various pathways related to amino acids, carbohydrates, and fatty acid metabolism to highlight the relationships among these metabolites (Figure 3).

The THP-1 cell line used in this study is a human leukemia monocytic cell line derived from the peripheral blood of a one-year-old patient with acute monocytic leukemia [18,19]. This cell line expresses a high level of citric acid (organic acid), myo-inositol (sugars and sugar alcohols), oleamide, alpha-palmitin, and cholesterol (fatty acids and lipids), but lower levels of saccharide, phosphoric acid, succinic acid, and myristic acid than do HSCs (Figure S1). The human monoblastic leukemia cell line U937, which is a valuable model for analyzing monocyte–macrophage differentiation, was isolated from the histiocytic lymphoma of a 37-year-old male patient and harbors the t(10;11)(p13;q14) translocation [20,21]. This cell line expresses higher levels of citric acid (organic acid), myo-inositol (sugars and sugar alcohols), oleamide, alpha-palmitin, and cholesterol (fatty acids and lipids), but lower levels of glucose, saccharide, cortisol, myristic acid, succinic acid, phosphoric acid, pyruvic acid, and lysine than do HSCs (Figure S2). It was possible to differentiate THP-1 and U937 monocytic circulatory leukemic cells, based on having similar abnormalities in the 11q23 translocation, into various types of macrophages or dendritic cells in vitro [22,23]. The basic difference between the two cell types is their origin and maturity. Since they can differentiate into tissues, they are more mature, whereas THP-1 cells are less mature as they originate from leukemic cells [19]. THP-1 and U937 as monocytic leukemia cells specifically expressed alpha-palmitin, and saccharides were relatively overexpressed in both cell lines compared to that of other leukemia cell lines. When THP-1 and U937 were compared to each other, THP-1 expressed more citric acid, hydroxylamine, oleamide, lysine, and oleic acid.

The KG-1 cell line was established from bone marrow cells of a patient with erythroleukemia evolving to AML with considerable pleomorphism with a predominance of myeloblasts and promyelocytes, and it harbors a partial hexasomy of the long arm of chromosome 8 [24]. This cell line expresses high levels of citric acid (organic acid), myo-inositol (sugars and sugar alcohols), oleamide, alpha-palmitin, cholesterol (fatty acids and lipids), and 5-oxo-proline (amino acids) compared to the levels produced by HSCs (Figure S3).

The human chronic myeloid leukemia K562 cell line is the first human immortalized erythroleukemia cell line established from a 53-year-old female CML patient [25,26]. Unlike CML, AML cell lines showed abnormal growth of undifferentiated and nonfunctional hemocytoblasts (leukemia blasts). However, in CML, cells carrying the Philadelphia chromosome express the Bcr-Abl fusion protein, are relatively mature, and have excessively accumulated abnormal white blood cells [27]. K562 highly expressed citric acid and malic acid, myo-inositol (sugars and sugar alcohols), oleamide, alpha-palmitin, cholesterol (fatty acids and lipids), 5-oxo-proline, beta-alanine, glycine, and aspartic acid (amino acids) compared to the levels produced by HSCs (Figure S4). Furthermore, they expressed at relatively higher levels myo-inositol, fructose, malic acid, glucose, cholesterol, 5-oxo-proline, beta-alanine, and citric acid metabolites compared to those produced by AML cell lines (THP-1, U937, and KG1). Especially, K562 was confirmed to have increased amino acid metabolites expression compared to that of AML.

To analyze the specific metabolomes of leukemia cells, this study used CD34+ cells extracted from human cord blood as normal control. CD34+ HSCs are precursors for producing all blood cell types and feature self-renewal and differentiation. One of the many proposed causes of leukemia is that HSCs can accumulate multiple mutations within a short period, and the existing ability for asymmetric differentiation and self-renewal can result in carcinogenic mutation [28,29]. Since normal HSCs and leukemic cells share self-renewal and diverse developmental pathways, HSCs with accumulated genetic variation are highly likely to be the origin of leukemia [30]. Hence, comparing and analyzing metabolism in HSCs and leukemia cells can help identify metabolic processes unique to each cell type and reveal the different mechanisms behind their differentiation and self-renewal. This, in turn, would provide important information for the development of drugs targeting leukemia metabolism. From our data, we infer that HSCs produce higher levels of succinic acid/serine/glucose/saccharide/palmitic acid/oleic acid/stearic acid than do leukemia cell lines. In contrast to a previous study in which fatty acid oxidation was critical for the growth of acute leukemia cells, the findings of our study reveal that fatty acid biosynthesis is downregulated in leukemia cell lines compared to that in HSCs [31,32].

Similar to a previous report, our metabolite profiling study also revealed that leukemia cell lines produce an overall higher number of metabolites compared to that of HSCs, possibly owing to a high rate of aerobic glycolysis associated with cancer cells [33,34]. Both glycolysis and OXPHOS are activated in leukemia cells owing to the enhanced need for energy metabolism and synthesis of intermediates to support cancer occurrence and development. Although these study results have helped to improve the understanding of leukemia cell metabolism, no new metabolic program for controlling the start and progress of leukemia could be suggested. Therefore, further studies focusing on multiple metabolic pathways using various systems and approaches are warranted to understand the alterations in metabolism and propose reliable biomarkers.

## 4. Materials and Methods

### 4.1. Chemicals and Reagents

Analytical grade methanol and water were purchased from Fisher Scientific (Pittsburgh, PA, USA). Pyridine, methoxyamine hydrochloride, *N*-methyl-*N*-(trimethylsilyl) trifluoroacetamide (MSTFA), and standard compounds were obtained from Sigma Chemical Co. (St. Louis, MO, USA).

### 4.2. Cell Culture

Normal human cord blood was provided by Soonchunhyang University Bucheon/Cheonan Hospital in South Korea. This study was approved by local Institutional Review Boards (2018-05-037-002). Fully informed consent was obtained from all patients before donation. Mononuclear cells were isolated by density gradient centrifugation over Ficoll-Plaque Plus (GE Healthcare, Marlborough, MA, USA) according to the manufacturer’s protocol. CD34^+^ cells were obtained through immunomagnetic selection (Miltenyi Biotec, Auburn, CA, USA) over two sequential columns. This procedure yielded CD34^+^ cells with 90–98% purity, which were then cultured in RPMI-1640 medium containing 10% fetal bovine serum (FBS), 100 ng/mL stem cell factor (SCF), thrombopoietin (TPO), and FMS-like tyrosine kinase 3 ligand (FLT3L) to expand cell numbers [35]. Leukemia cell lines (THP-1, KG-1, HL-60, U-937, and K562) were purchased from the ATCC (Manassas, VA, USA). Cell lines were cultured in RPMI1640 medium supplemented with 10% heat-inactivated FBS and 1% penicillin/streptomycin (Invitrogen) at 37 °C in a humidified incubator maintained at 5% CO_2_.

### 4.3. Sample Collection and Preparation for Metabolite Analysis

Metabolites were extracted from leukemia cell lines as described by He et al. [14] with some modifications. Briefly, cell samples were extracted with 100% methanol (1 mL) and 10 µL internal standard solution (2-chlorophenylalanine, 1 mg/mL in water) using an MM400 mixer mill (Retsch^®^, Haan, Germany) at a frequency of 30 s^−1^ for 10 min, followed by 10 min of sonication. Subsequently, the extracted samples were centrifuged at 10,000 rpm for 10 min at 4 °C, and the supernatants were filtered using 0.2-µm polytetrafluorethylene (PTFE) filters (Chromdisc, Daegu, Korea). The filtered supernatants were completely dried using a speed vacuum concentrator (Biotron, Seoul, Korea). The final concentration of the analyzed sample was 10 mg/mL.

### 4.4. Gas Chromatography–Time-of-Flight Mass Spectrometry Analysis

Gas chromatography-time-of-flight mass spectrometry (GC-TOF-MS) analysis was performed using an Agilent 7890A gas chromatograph system coupled with an Agilent 7693 autosampler (Agilent, Atlanta, GA, USA) as previously described [15]. For analysis, all dried samples were oximated with 50 µL of methoxyamine hydrochloride (20 mg/mL in pyridine) for 90 min at 30 °C and silylated with 50 µL of MSTFA for 30 min at 37 °C. The derivatized sample (1 µL) was injected into the GC-TOF-MS instrument in the split-less mode. The temperatures of the injector and ion source were maintained at 250 °C and 230 °C, respectively. The column temperature was sustained at 75 °C for 2 min and then raised to 300 °C at 15 °C/min and subsequently maintained for 3 min. The acquisitions were recorded at the rate of 10 scans/s with a mass scan range of 50–1000 *m*/*z*. The GC-TOF-MS analysis was performed with three repetitive chromatographic runs for each sample extracts. Discriminant metabolites were identified by comparing the retention times and mass fragment patterns with those of standard compounds, the NIST database (version 2.0, 2011, FairCom, Gaithersburg, MD, USA), and an in-house library.

### 4.5. Data and Statistical Analysis

MS data processing and multivariate statistical analysis were conducted as previously described [15]. Significantly different metabolites derived from GC-TOF-MS data were tentatively identified using standard compound retention time and MS fragments. Moreover, we confirmed the MS spectrum data for selected metabolites with in-house libraries and available web databases, including Wiley 9, the NIST database (Version 2.0, 2011 FairCom; Gaithersburg, MD, USA), and the Human Metabolome Database (HMDB; http://www.hmdb.ca/). Statistical analysis was performed using PASW Statistics (IBM SPSS Inc., Chicago, IL, USA). The significantly discriminant metabolites from the analytical datasets were selected based on the variable importance in projection, VIP > 0.7 at *p* < 0.05. Further, the significant differences (*p* value < 0.05) among the selected metabolites were evaluated through one-way ANOVA using STATISTICA 7 (Stat Soft Inc., Tulsa, OK, USA). Results with *p* < 0.05 were considered statistically significant.

## 5. Conclusions

In conclusion, based on our results, we confirmed that the AML and CML cell lines analyzed in this study have higher overall metabolic activity than do HSCs, which may be attributed to the accumulation of chromosomal abnormalities. Compared to HSCs, we confirmed specifically increased expression of the citric acid, myo-inositol, oleamide, alpha-palmitin, and cholesterol metabolites in leukemia cell lines. These results provide information that may help identify leukemia cell-specific metabolites and related mechanisms in future studies.

## Figures and Tables

**Table 1:** List of the significantly different metabolites selected using the PLS-DA model based on the GC-TOF-MS dataset for 4 different leukemia cells and normal HSC.

No.	Ret (min) ^a^	VIP1	VIP2	Tentative Identifications ^b^	Unique Mass (*m*/*z*)	MS Fragment Pattern (*m*/*z*)	REF ^c^
Organic acids							
1	5.13	0.78	0.51	Lactic acid	117	73, 147, 117, 75, 66, 59, 148, 191	STD/MS
2	5.96	1.34	1.04	Pyruvic acid	220	73, 147, 100, 133, 59, 72, 86, 220	STD/MS
3	7.61	1.80	1.27	Succinic acid	247	73, 147, 75, 247, 59, 77, 69, 50	STD/MS
4	9.19	0.35	1.67	Malic acid	233	73, 147, 55, 75, 52, 133, 156, 233	STD/MS
5	11.76	0.78	1.71	Citric acid	273	73, 147, 75, 273, 74, 50, 149, 133	STD/MS
Amino acids							
6	7.59	1.29	1.11	Glycine	174	73, 174, 147, 341, 86, 59, 77, 100	STD/MS
7	8.08	1.61	1.15	Serine	204	73, 100, 204, 119, 188, 218, 193	STD/MS
8	8.33	0.80	1.37	Threonine	219	73, 58, 174, 57, 147, 75, 86, 219	STD/MS
9	8.66	1.01	1.55	β-Alanine	248	73, 174, 147, 248, 86, 59, 100, 133	STD/MS
10	9.45	1.09	1.08	Aspartic acid	232	73, 156, 232, 147, 100, 75, 79, 52	STD/MS
11	9.51	1.17	1.49	5-oxo-proline	156	156, 73, 147, 75, 59, 230, 258	STD/MS
12	11.73	1.57	1.11	Ornithine	142	73, 142, 174, 147, 59, 74, 86, 100	STD/MS
13	12.42	1.16	1.30	Lysine	156	73, 75, 147, 59, 174, 156, 103	STD/MS
Sugars and sugar alcohols							
14	12.18	0.79	1.56	Fructose	217	73, 103, 217, 147, 74, 307, 133, 117	STD/MS
15	12.37	1.56	1.26	Glucose	160	73, 147, 205, 160, 103, 319, 74, 129	STD/MS
16	12.63	1.86	1.23	Saccharide 1	319	73, 147, 103, 217, 205, 319, 117, 129	MS
17	13.20	1.09	1.49	Saccharide 2	204	73, 204, 147, 75, 117, 217, 205, 129	MS
18	13.62	0.89	1.67	myo-Inositol	217	73, 147, 217, 191, 305, 129, 133	STD/MS
Fatty acids and lipids							
19	11.51	1.56	1.12	Phosphorylethanolamine	299	73, 100, 59, 299, 172, 147, 74, 114	MS
20	11.84	1.69	1.13	Myristic acid	285	73, 75, 117, 129, 132, 55, 145, 131	STD/MS
21	13.14	1.80	1.21	Palmitic acid	313	73, 75, 117, 132, 129, 55, 145, 131	STD/MS
22	14.16	1.47	0.98	Linoleic acid	337	75, 73, 67, 55, 81, 79, 129, 117, 337	STD/MS
23	14.19	1.70	1.36	Oleic acid	339	75, 73, 55, 117, 129, 67, 145, 339	STD/MS
24	14.33	1.84	1.27	Stearic acid	341	73, 75, 117, 132, 129, 131, 145, 341	STD/MS
25	16.21	1.47	1.55	α-Palmitin	371	73, 57, 55, 147, 75, 69, 129, 371	MS
26	19.74	1.35	1.26	Cholesterol	129	129, 73, 75, 55, 57, 81, 95, 105	STD/MS
Electron Transport Chains							
27	5.65	1.15	0.91	Hydroxylamine	146	73, 133, 146, 59, 119, 86, 147, 130	STD/MS
28	7.31	1.65	1.11	Phosphoric acid	299	73, 299, 133, 211, 300, 207, 193	STD/MS
29	7.56	0.90	0.65	Cortisol	256	73, 107, 77, 55, 256, 69, 84, 140	STD/MS
Etc.							
30	6.35	1.03	1.50	N.I. 1	184	73, 58, 69, 228, 110, 77, 134, 184	‒
31	6.66	1.07	0.77	N.I. 2	228	73, 69, 58, 228, 110, 77, 134, 184	‒
32	7.87	0.89	0.71	N.I. 3	184	73, 184, 134, 59, 77, 86, 100, 69	‒
33	9.14	1.33	1.58	N.I. 4	281	73, 147, 281, 327, 74, 282, 59, 415	‒
34	10.48	1.32	1.61	N.I. 5	355	73, 355, 147, 221, 281, 74, 356	‒
35	11.63	1.49	1.69	N.I. 6	429	73, 147, 221, 429, 74, 355, 207	‒
36	12.66	1.44	1.67	N.I. 7	281	73, 147, 281, 221, 74, 207, 282, 341	‒
37	13.43	1.15	0.82	N.I. 8	136	55, 69, 122, 56, 54, 67, 83, 136	‒
38	14.45	1.43	1.59	N.I. 9	355	73, 147, 221, 355, 281, 429, 207	‒
39	15.76	1.43	1.08	N.I. 10	55	55, 69, 57, 83, 54, 56, 67, 122	‒
40	15.97	1.49	1.56	N.I. 11	355	73, 147, 221, 281, 355, 207, 429	‒
41	16.66	1.53	1.59	N.I. 12	221	73, 147, 221, 355, 281, 207, 429	‒
42	17.30	1.50	1.61	N.I. 13	221	73, 147, 221, 281, 355, 207, 74	‒
43	17.35	1.12	0.99	N.I. 14	131	75, 131, 55, 144, 116, 128, 69, 394	‒

^a^ Retention time; ^b^ Tentative identifications based on variable importance to projection (VIP) > 0.7 and *p* value < 0.05; ^c^ Metabolites identified based on the in-house library of standard compounds (STD/MS: Standard compounds/Mass spectrometry fragments).

## Supplementary Materials

The following are available online at https://www.mdpi.com/2218-1989/10/11/427/s1, Figure S1: Differential metabolites identified in THP-1 compared with those from HSCs, Figure S2: Differential metabolites identified in U937 compared with those from HSCs, Figure S3: Differential metabolites identified in KG-1 compared with those from HSCs, Figure S4: Differential metabolites identified in K562 compared with those from HSCs.
