# Supplementary figures and images for: Profiling of Metabolic Differences between Hematopoietic Stem Cells and Acute/Chronic Myeloid Leukemia
Source: Metabolites. 2020 Oct 26;10(11):427. doi: 10.3390/metabo10110427 (PMC7692247; doi:10.3390/metabo10110427)

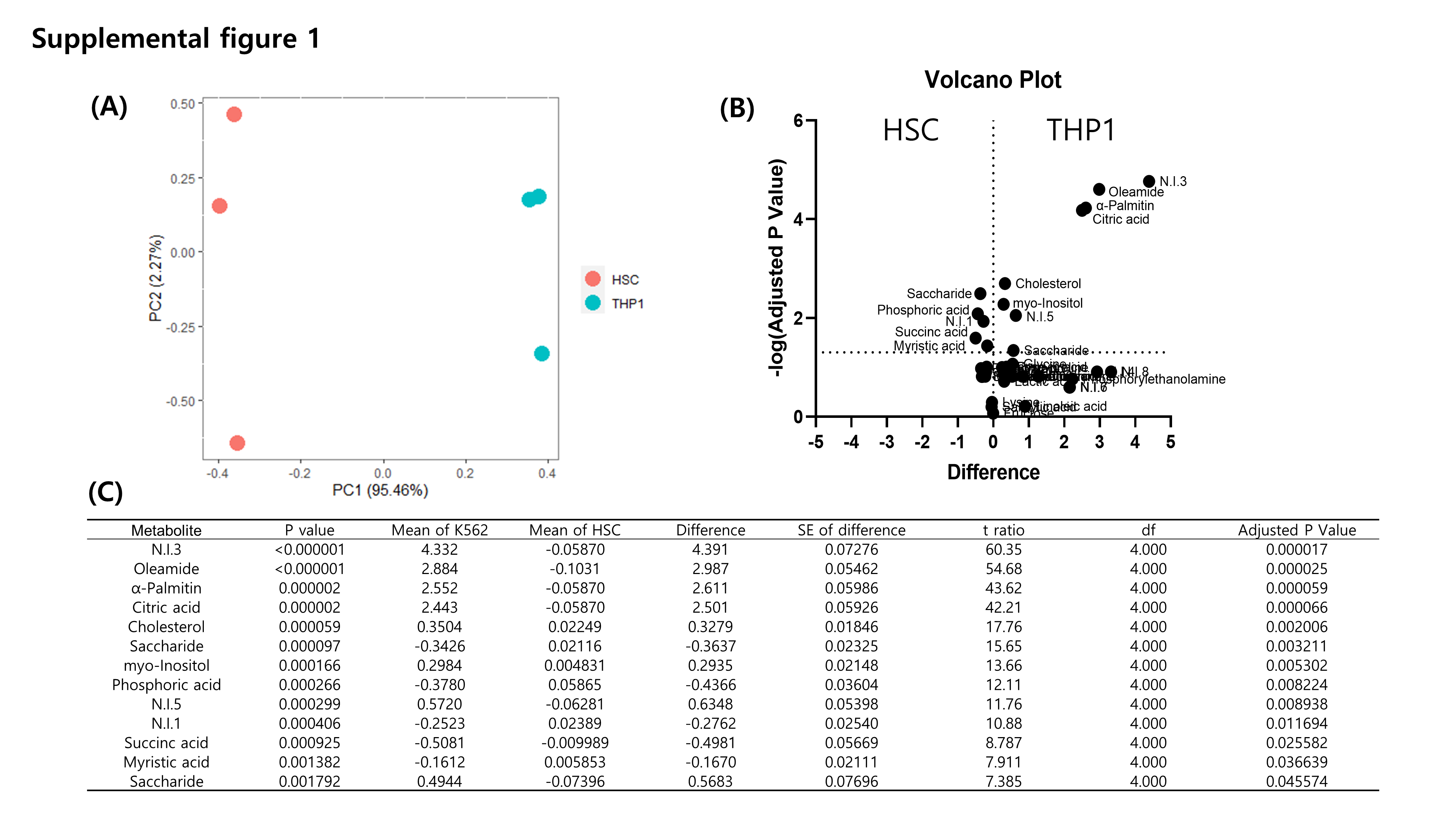

Supplement: Supplementary file 1 [file metabolites-10-00427-s001.zip › Song et al (Supplement figure 1).TIF]

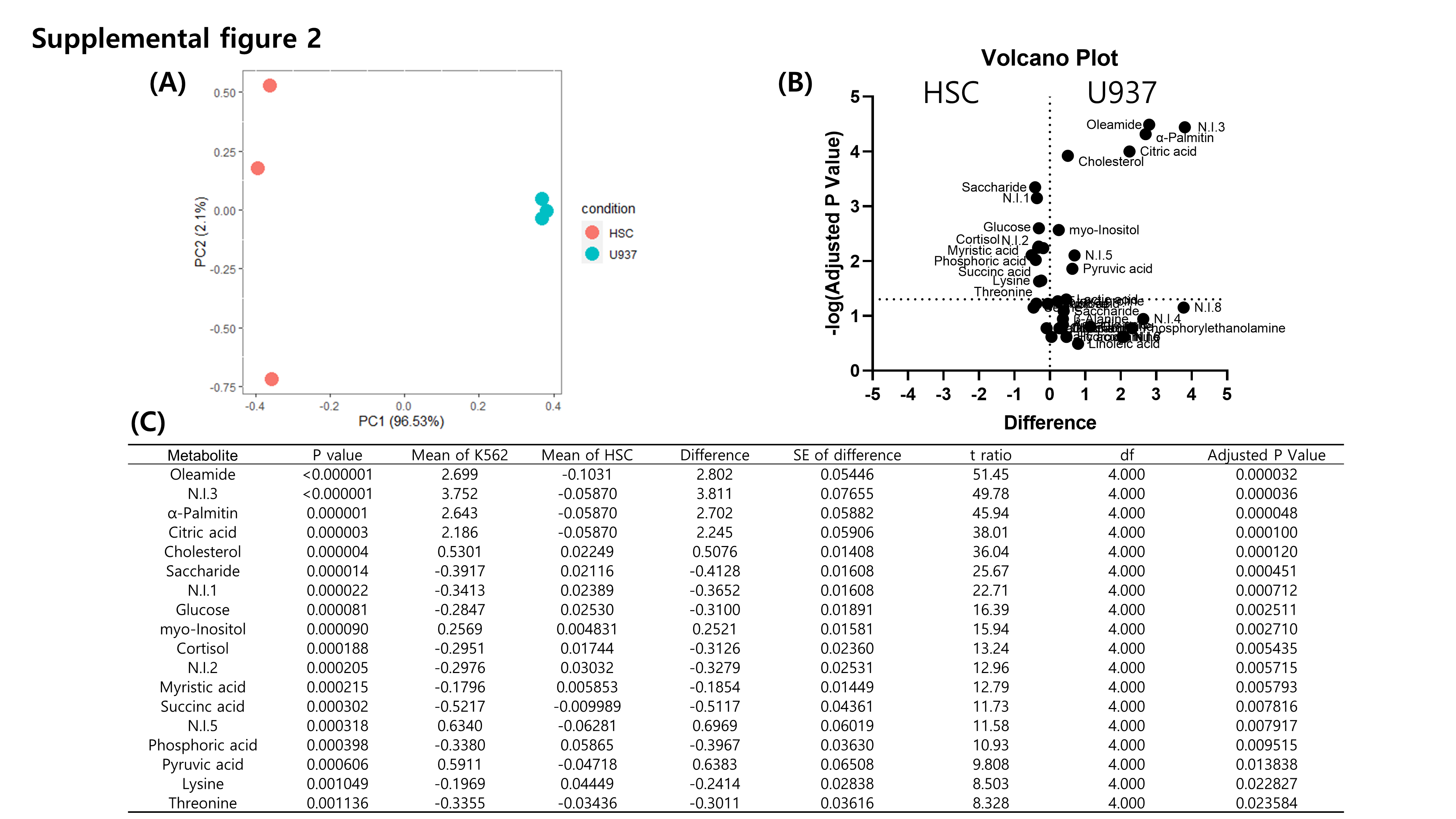

Supplement: Supplementary file 1 [file metabolites-10-00427-s001.zip › Song et al (Supplement figure 2).TIF]

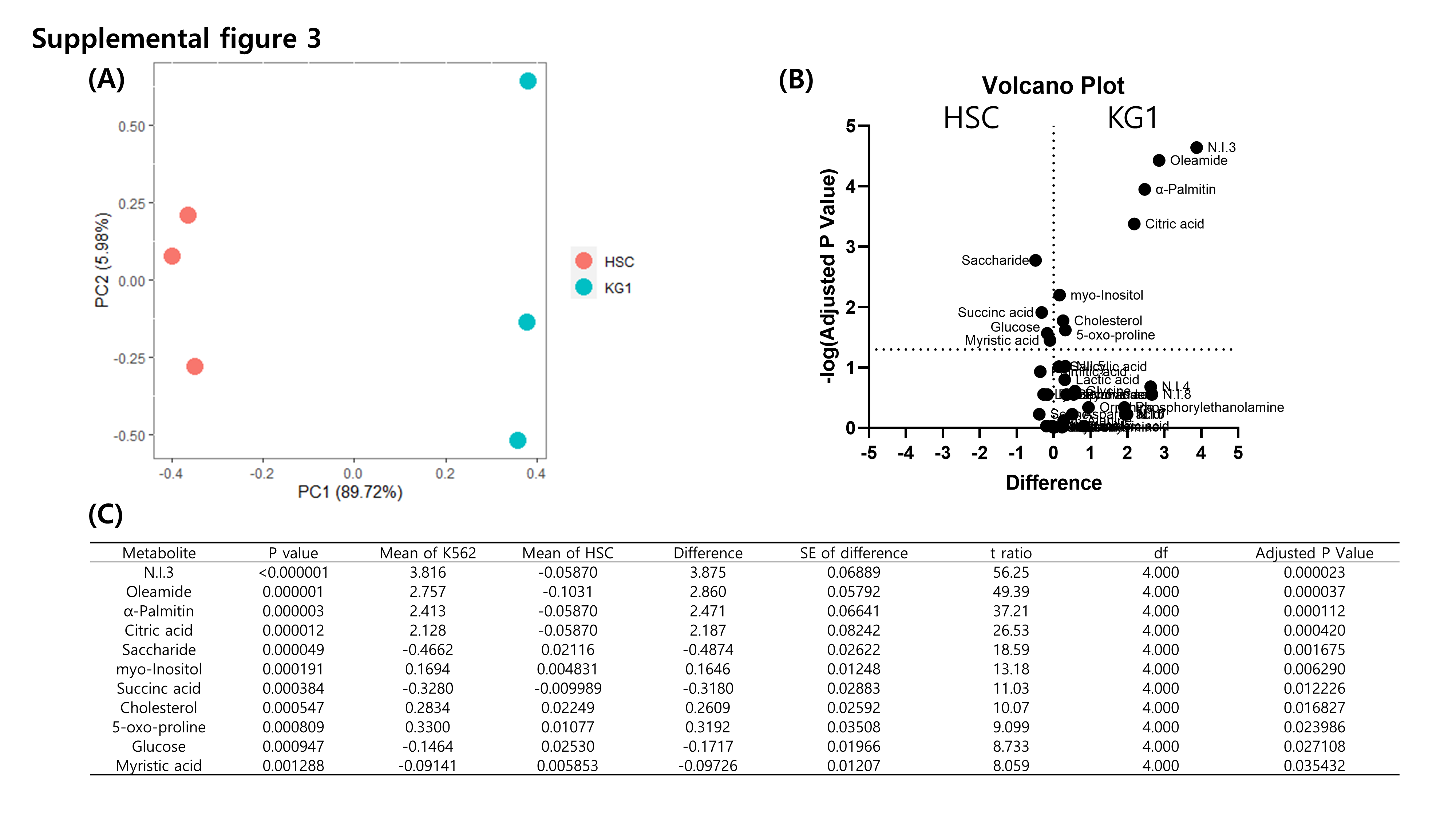

Supplement: Supplementary file 1 [file metabolites-10-00427-s001.zip › Song et al (Supplement figure 3).TIF]

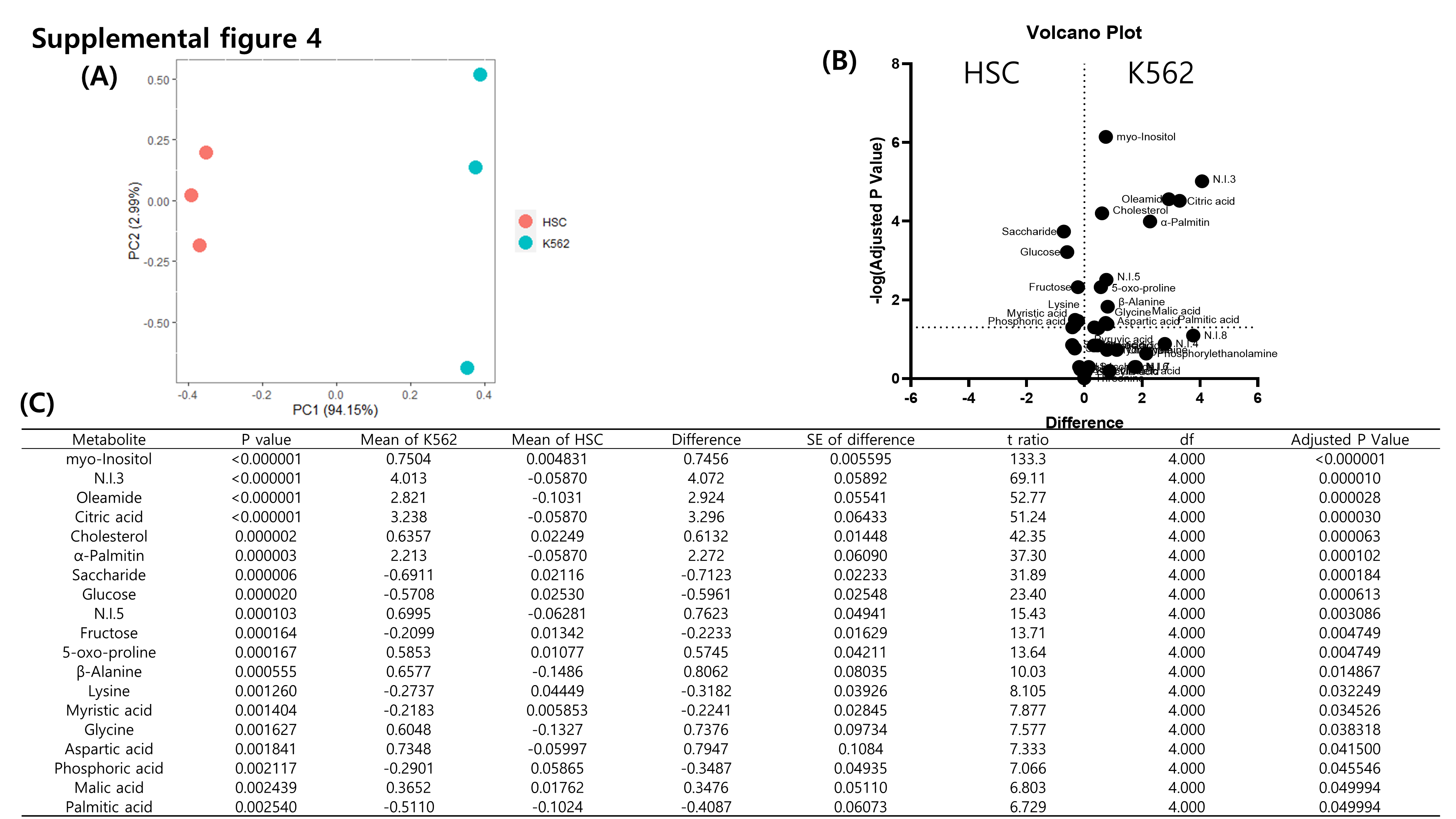

Supplement: Supplementary file 1 [file metabolites-10-00427-s001.zip › Song et al (Supplement figure 4).TIF]
